# Supplementary material for: KLF-1 orchestrates a xenobiotic detoxification program essential for longevity of mitochondrial mutants
Source: Nat Commun. 2019 Jul 25;10:3323. doi: 10.1038/s41467-019-11275-w (PMC6658563; doi:10.1038/s41467-019-11275-w)
Supplement: Supplementary file 1 — Supplementary Information [file 41467_2019_11275_MOESM1_ESM.pdf]

KLF-1 orchestrates a xenobiotic detoxification program essential for longevity of mitochondrial mutants

Herholz et al.

Supplementary Figure 1

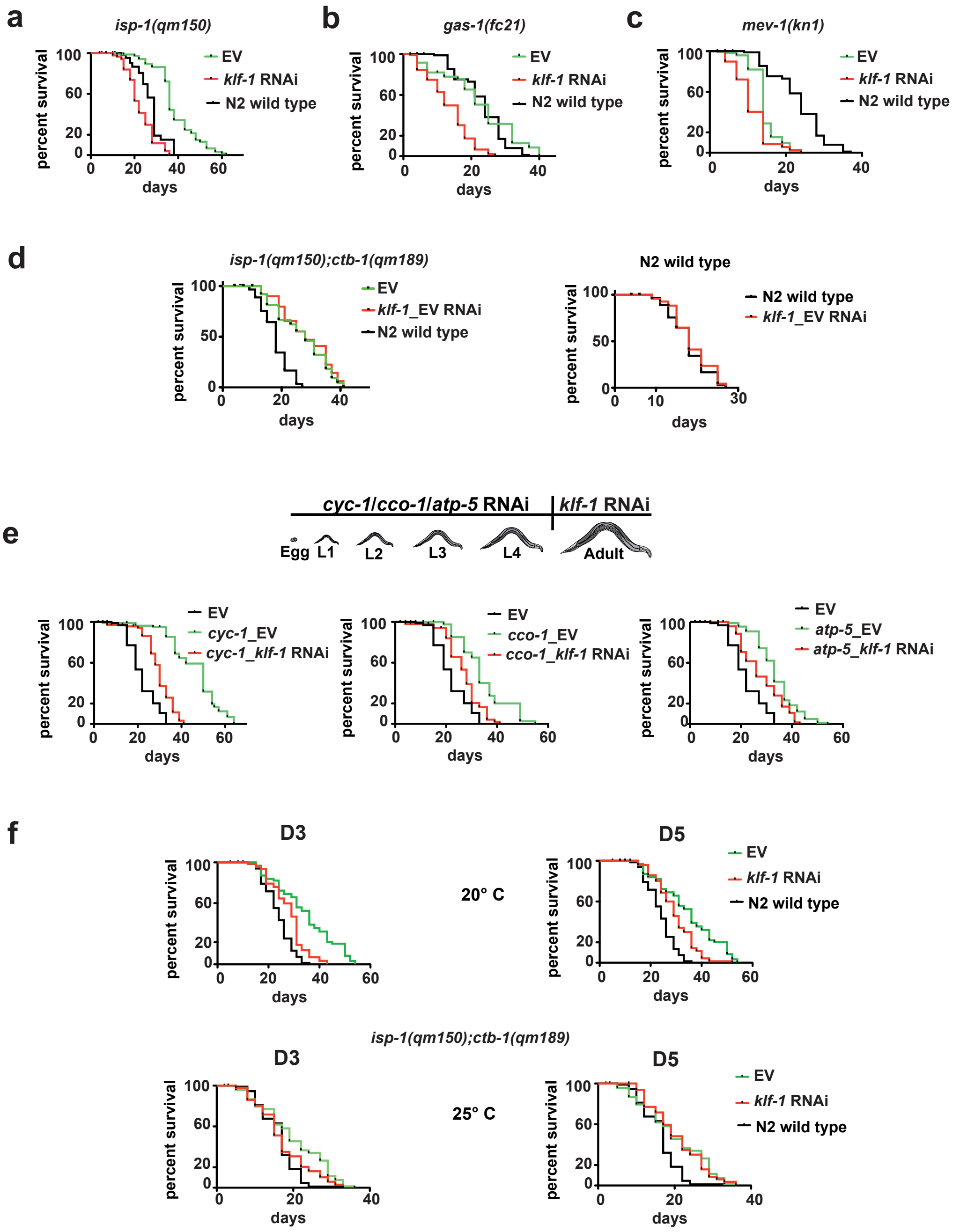

Supplementary Figure 1. KLF-1 activity in early adulthood is crucial for *isp-1;ctb-1* longevity. (**a**) Lifespan curve of complex III *isp-1(qm150)* mutant on control (EV) or *klf-1* RNAi. (**b-c**) *klf-1* knockdown in *gas-1(fc21)* (complex I) and *mev-1(kn1)* (complex II) mutant shortens lifespan. (**d**) Lifespan curves of *isp-1(qm150);ctb-1(qm189)* (left) and N2 wild type animals (right) grown on control or *klf-1* RNAi for two subsequent generations. The animals from *klf-1* RNAi plates of the second generation were then transferred to control RNAi at L4 larval stage. (**e**) Lifespan curves of N2 wild type animals grown either on complex III (*cyc-1*), complex IV (*cco-1*) and complex V (*atp-5*) RNAi during developmental stages and then transferred to either control (EV) or *klf-1* RNAi plates. (**f**) Survival curve of *isp-1(qm150);ctb-1(qm189)* animals grown on control RNAi plates until third (D3) or fifth (D5) day of adulthood and then transferred to *klf-1* RNAi plates at 20°C degrees (upper panel) and 25°C degrees (lower panel).

Supplementary Figure 2

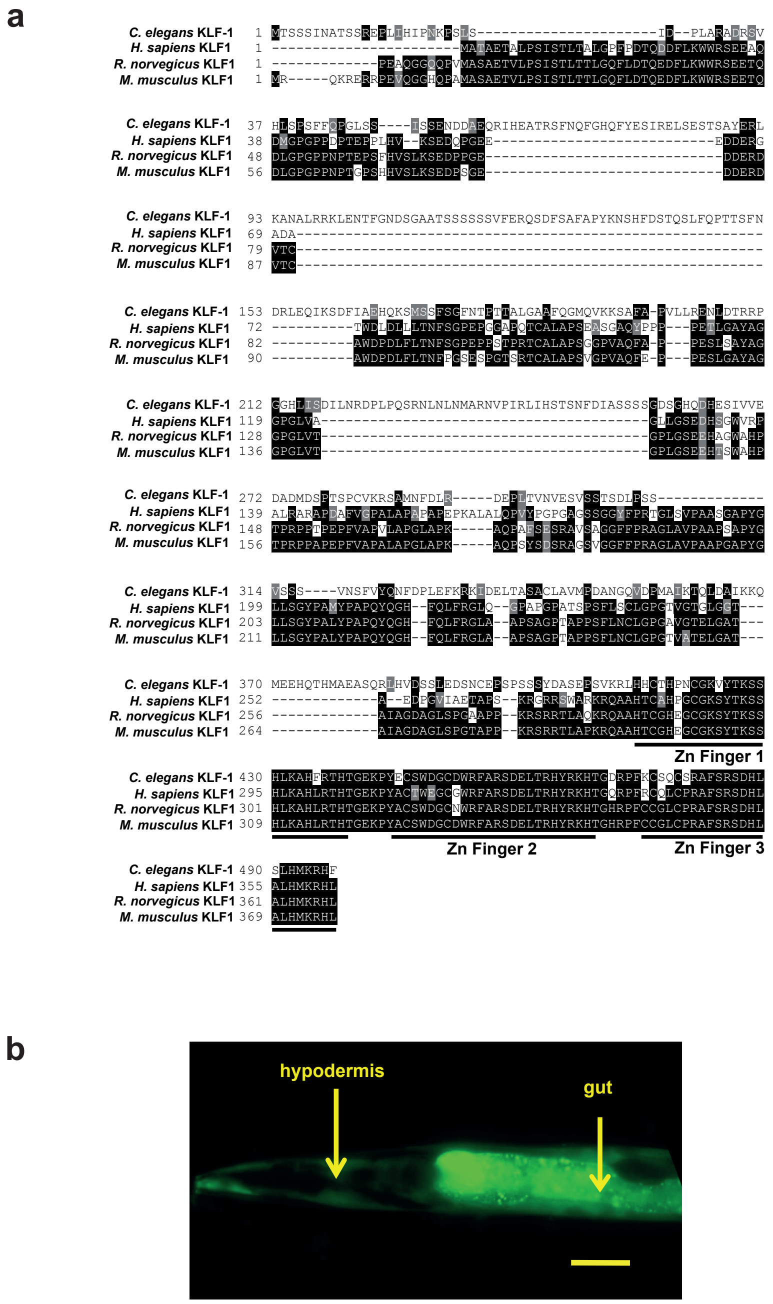

Supplementary Figure 2. KLF-1 shares homology with mammalian KLFs in its Zn-finger domain. (a) Alignment of *C. elegans* KLF-1 with KLF1 sequences of *H. sapiens*, *R. norvegicus*, *M. musculus*. (**b**) Fluorescent image of a WT worm expressing *gfp* under the *klf-1* promoter. Yellow arrows indicate tissues where strong gfp expression is observed. Scale bar 20μm.

Supplementary Figure 3

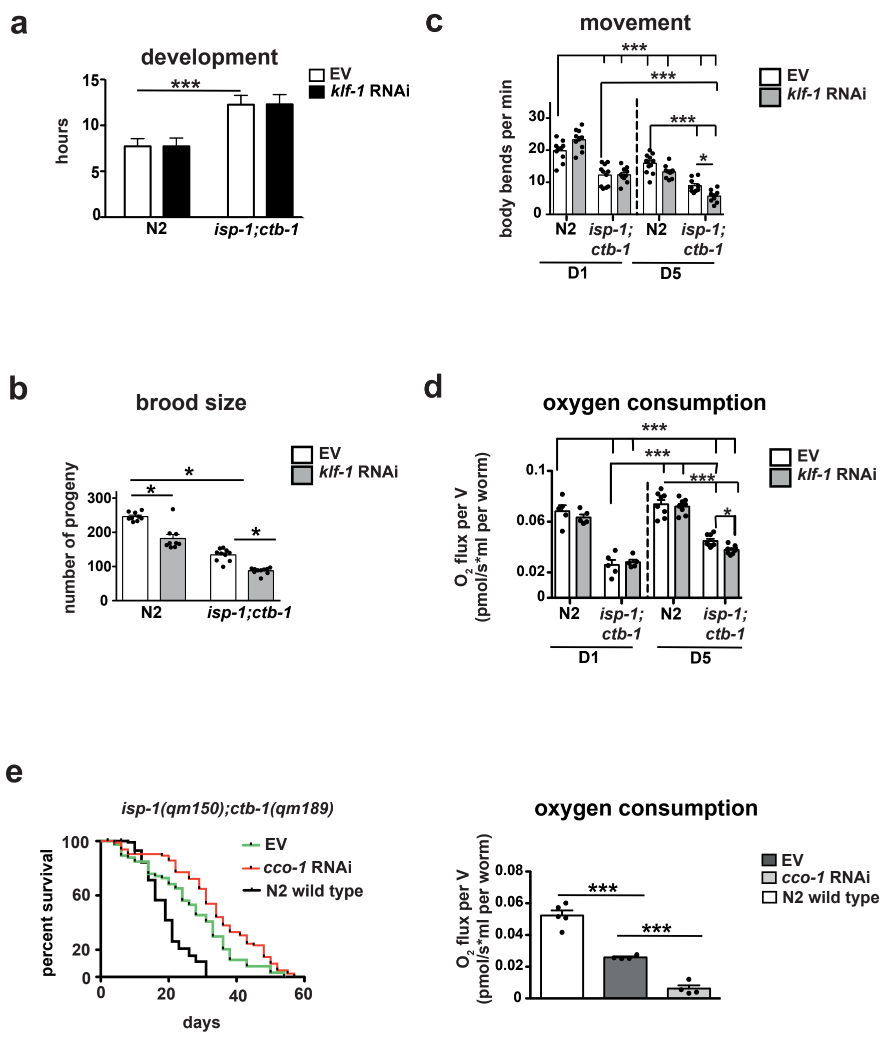

Supplementary Figure 3. KLF-1 has mild effects on behavioural phenotypes of *isp-1;ctb-1*. (**a**) Development analysis of N2 wild type and *isp-1(qm150);ctb-1(qm189)*. Developmental time was assayed that animals needed to reach adulthood starting from eggs on. n=300 animals were analyzed on control (EV) and *klf-1* RNAi. Brood size (**b**) and movement rates (**c**) were measured in N2 wild type and *isp-1(qm150);ctb-1(qm189)* animals on control (EV) or *klf-1* RNAi. n=10 animals were assayed per condition. (**d**) Oxygen consumption in N2 wild type and *isp-1(qm150);ctb-1(qm189)* animals either on control (EV) or *klf-1* RNAi was analyzed at the first (D1) or the fifth (D5) day of adulthood. Graph represents data from n=5 replicates, each with 300 animals. (e) Lifespan curve of *isp-1(qm150);ctb-1(qm189)* mutants exposed to *cco-1* RNAi during whole life (left) and oxygen consumption rates in the same animals at D1. Graph represents data from n=4 replicates, each with 300 animals. For all panels, data are presented as mean±SEM. $*p<0.05$, $***p<0.001$, One-way ANOVA with Tukey post hoc test.

Supplementary Figure 4

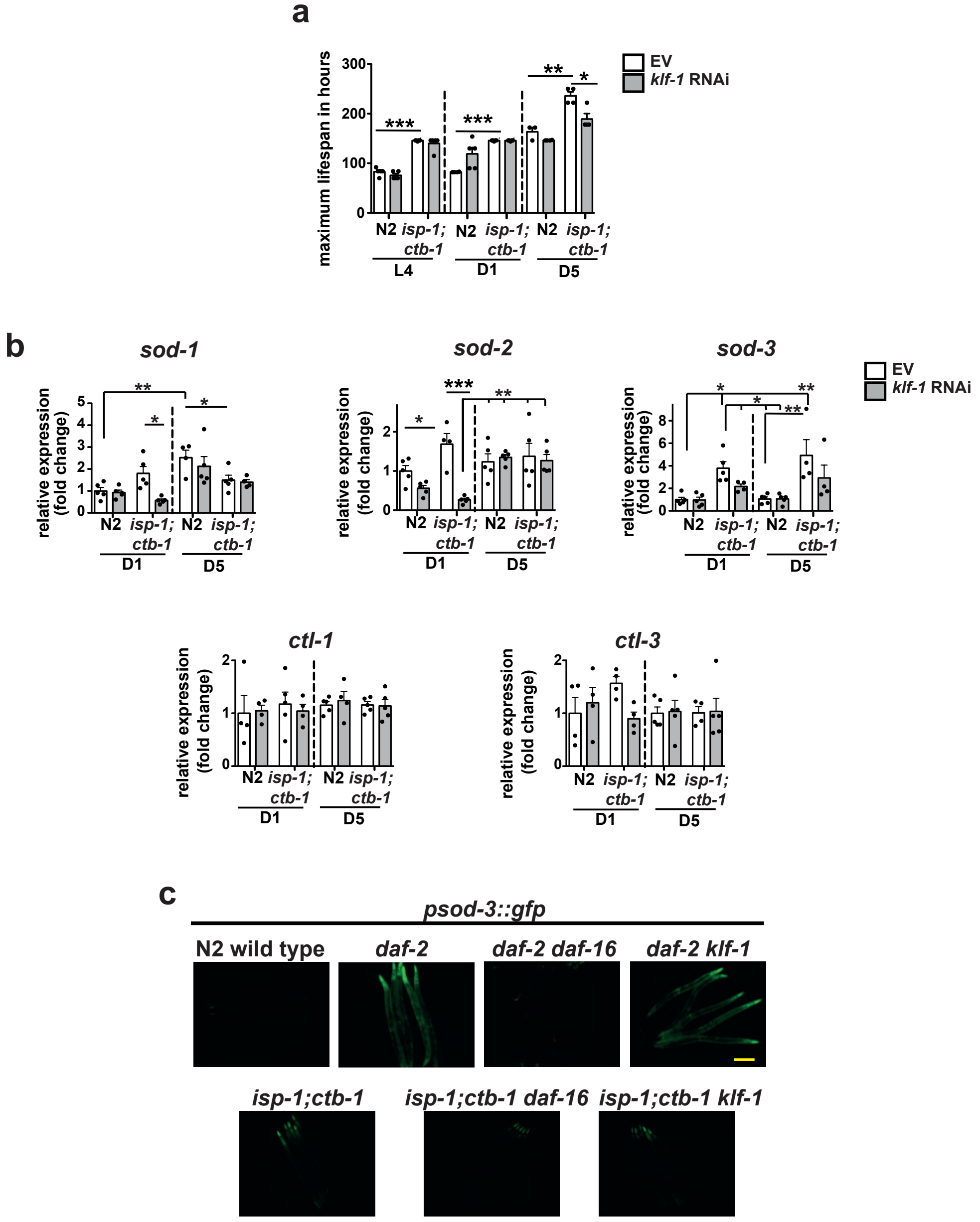

Supplementary Figure 4. KLF-1 does not directly regulate the expression of the oxidative stress response genes. (**a**) Paraquat sensitivity was assayed as survival rates on 16mM paraquat. Animals were exposed to paraquat either at L4 larval stage, the first (D1) or the fifth (D5) day of adulthood. For each condition, n=5 plates, each with 20 worms were used. (**b**) Expression of genes coding for proteins involved in the oxidative stress response, *sod-1*, *sod-2*, *sod-3, ctl-1* and *ctl-3* was analyzed by qPCR in N2 wild type and *isp-1(qm150);ctb-1(qm189)* mutant at the D1 or D5 of adulthood. Animals were grown on control (EV) or *klf-1* RNAi. For all panels, data are presented as mean±SEM. *p<0.05, **p<0.01, ***p<0.001, One-way ANOVA with Tukey post hoc test. n=5 samples per condition. (**c**) Fluorescent images of a strain expressing *gfp* under *sod-3* promoter. *psod-3::gfp* array was crossed into *daf-2(e1370)* and *isp-1(qm150);ctb-1(qm189)* mutants and animals were grown on control (EV), *daf-16* or *klf-1* RNAi until D1 when the pictures were taken. Scale bar 100µm.

Supplementary Figure 5

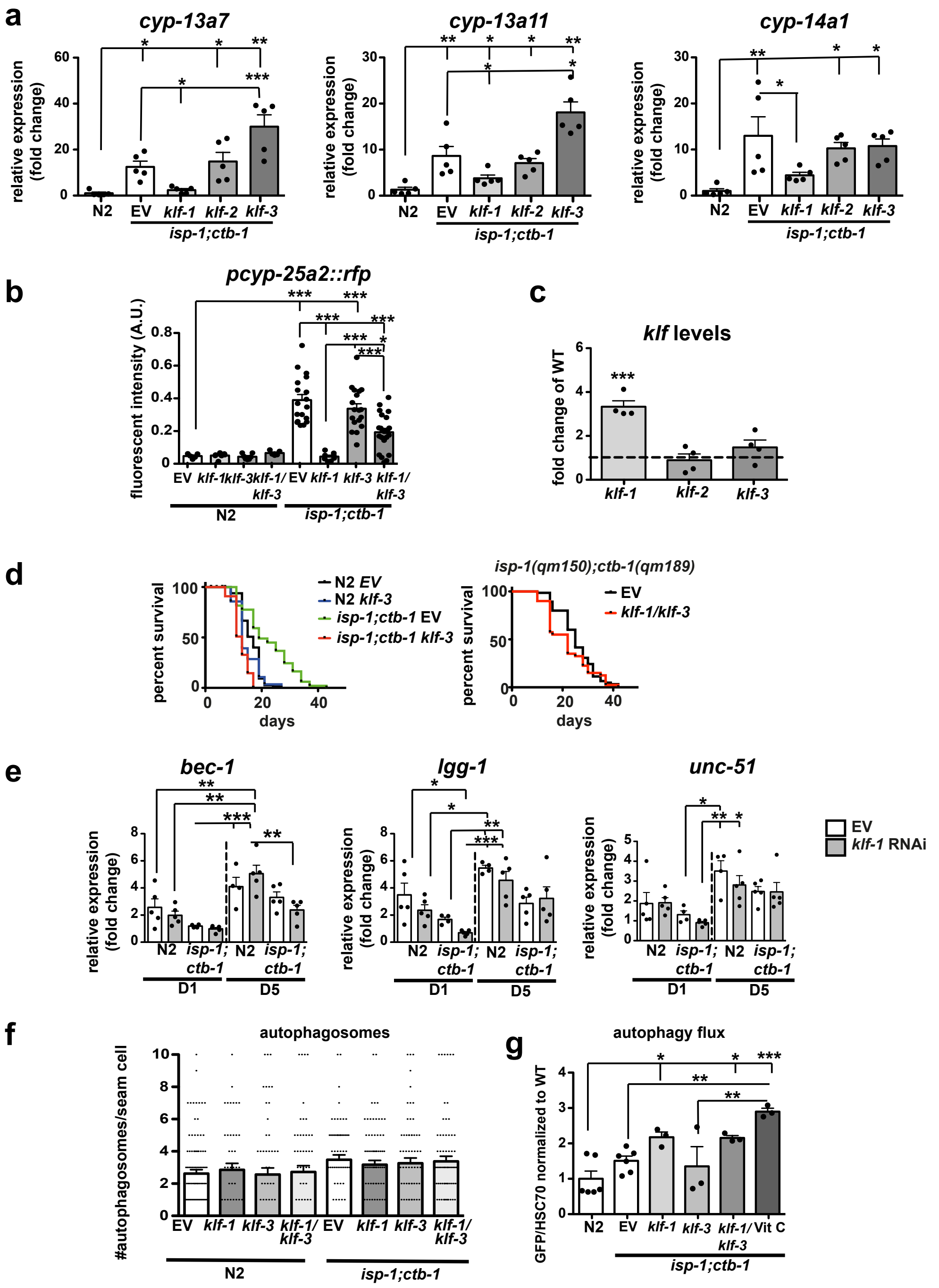

Supplementary Figure 5. KLF-2 and KLF-3 do not mediate *cyp* gene expression in *isp-1;ctb-1*. (**a**) *cyp-13a7*, *cyp-13a11* and *cyp-14a1* mRNA levels were assayed by qPCR in WT and *isp-1(qm150);ctb-1(qm189)* mutant at the first (D1) day of adulthood after exposure to either *klf-1*, *klf-2* or *klf-3* RNAi. n=5 independent samples were used per condition. *p<0.05, **p<0.01, ***p<0.001, One-way ANOVA with Tukey post hoc test. (**b**) Activity of the *pcyp-25a2::rfp* reporter in N2 wild type and *isp-1(qm150);ctb-1(qm189)* genetic backround. n=10 animals were imaged at fifth (D5) day of adulthood upon *klf-1*, *klf-3* or combined *klf-1* and *klf-3* RNAi. Data are presented as mean±SEM. *p<0.05, ***p<0.001, One-way ANOVA with Tukey post hoc test. (**c**) Expression level of *klf-1*, *klf-2* and *klf-3* in *isp-1(qm150);ctb-1(qm189)* mutant as analyzed by qPCR in *isp-1(qm150);ctb-1(qm189)* mutant at D1. For all panels, data are presented as mean±SEM. *p<0.05, **p<0.01, ***p<0.001, Student's T-test. n=4 independent samples. (**d**) Lifespan analysis of N2 wildtype and *isp-1(qm150);ctb-1(qm189)* animals grown on control (EV) or *klf-3* RNAi (left) or combined *klf-1* and *klf-3* RNAi (right). (**e**) *bec-1*, *lgg-1* and *unc-51* mRNA levels were assayed by qPCR in N2 wild type and *isp-1(qm150);ctb-1(qm189)* mutant at D1 and D5 in control (EV) or upon *klf-1* RNAi. n=5 independent replicates were used per condition. *p<0.05, **p<0.01, ***p<0.001, One-way ANOVA with Tukey post hoc test. (**f**)

Number of GFP positive puncta in the seam cells of L4 stage N2 wild type or *isp-1(qm150);ctb-1(qm189)* animals expressing *plgg-1::lgg-1-gfp*. Animals were grown on control (EV) or *klf-1*, *klf-3* or combined *klf-1* and *klf-3* RNAi. n=30 animals per condition. (**g**) Proteins were isolated from *plgg-1::lgg-1-gfp* carrying strain in N2 wild type and *isp-1(qm150);ctb-1(qm189)* genetic background, at the L4 stage. Western blots were probed against GFP antybody. Shown is quantification of the Western blots from n=3 independent replicates per condition. *p<0.05, **p<0.01, ***p<0.001, One-way ANOVA with Tukey post hoc test.

Supplementary Figure 6

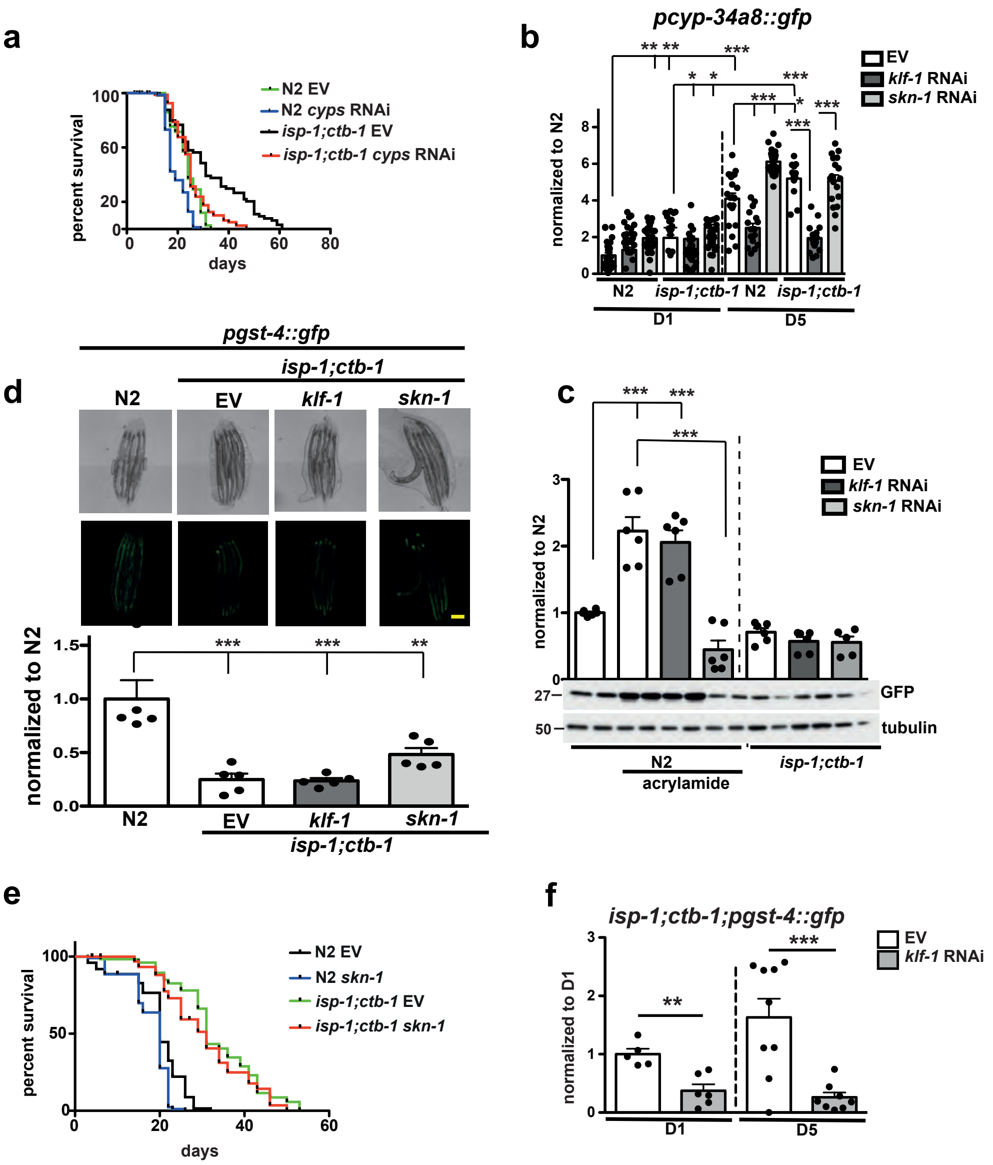

Supplementary Figure 6. Phase II response is not activated in *isp-1(qm150);ctb-1(qm189)*. (**a**) Lifespan analysis of N2 wild type and *isp-1(qm150);ctb-1(qm189)* animals grown on control or combined *cyp-25a1* and *cyp-13a11* RNAi (*cyps*). (**b**) Quantification of *gfp* expression under *cyp-34a8* promoter in N2 wild type and *isp-1(qm150);ctb-1(qm189)* mutant background upon *klf-1* and *skn-1* RNAi at the first (D1) or the fifth (D5) day of adulthood. Data are presented as mean±SEM. *p<0.05, **p<0.01, ***p<0.001, One-way ANOVA with Tukey post hoc test. n=15 animals. (**c**) Western blot showing GFP levels in the protein isolates from *pgst-4::gfp* strain from N2 wild type and *isp-1(qm150);ctb-1(qm189)* mutant background at D1. N2 animals were treated with acrylamide at D1. Data are presented as mean±SEM. ***p<0.001, One-way ANOVA with Tukey post hoc test. n=6 independent samples per condition. (**d**) Upper panel shows representative confocal images of *gfp* expressed under *gst-4* promoter in wild type and *isp-1(qm150);ctb-1(qm189)* animals grown on *klf-1* or *skn-1* RNAi at D1. Scale bar 100µm. Lower panel is the quantification of n=10 animals. *p<0.05, **p<0.01, One-way ANOVA with Tukey post hoc test. (**e**) Lifespan analysis of N2 wild type and *isp-1(qm150);ctb-1(qm189)* animals grown on control (EV) or *skn-1* RNAi. (**f**) Quantification of *gfp* expression under *gst-4* promoter in *isp-1(qm150);ctb-1(qm189)* mutant upon *klf-1* knockdown at the D1 or D5 of adulthood. Data are presented as mean±SEM. **p<0.01, ***p<0.001, Student T-test. n=5 animals per condition.

Supplementary Figure 7

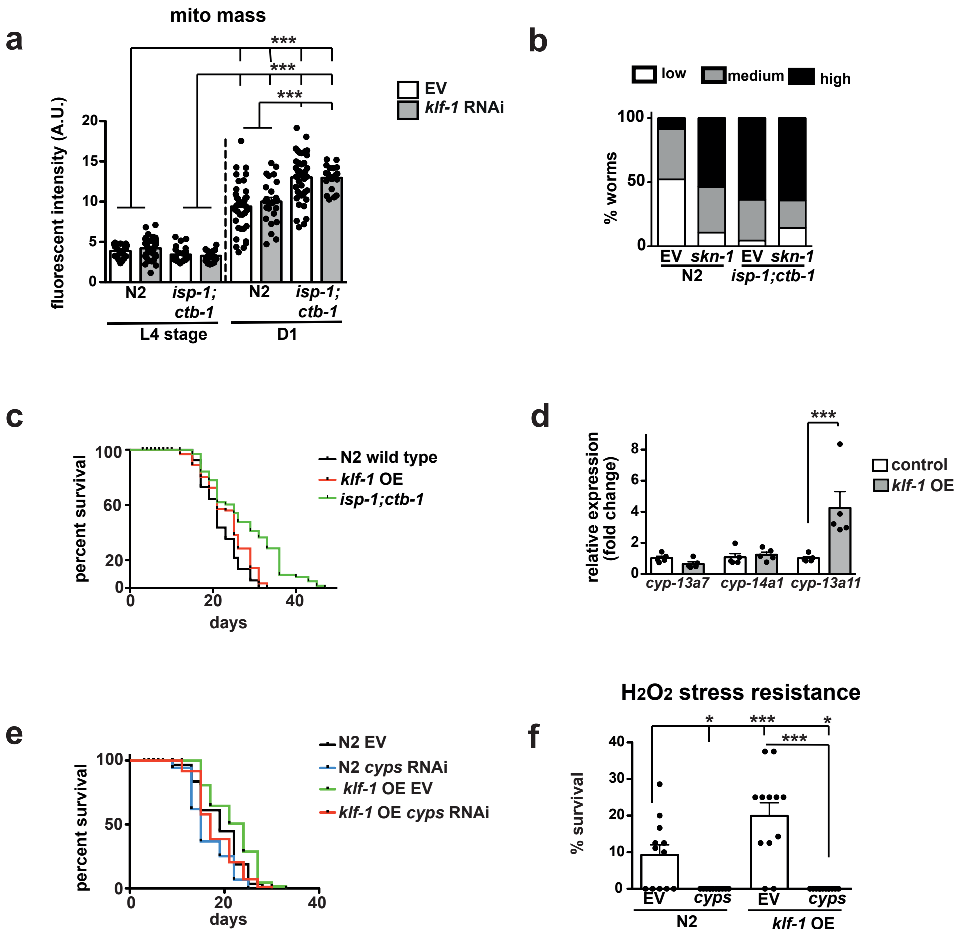

Supplementary Figure 7. KLF-1 overexpression extends lifespan of wild type animals. (**a**) N2 wild type and *isp-1(qm150);ctb-1(qm189)* animals were stained with Mitotracker Deep Red at L4 stage and first (D1) day of adulthood to assay the mitochondrial mass. Animals were grown either in control (EV) or *klf-1* RNAi. n=20 animals per condition. (**b**) Strain carrying *pvha-6::klf-1-yfp* array in N2 wild type and *isp-1(qm150);ctb-1(qm189)* genetic background was assay for nuclear translocation of KLF-1. Animals were grown either on control (EV) or *skn-1* RNAi and assayed at D1. n=10 animals. (**c**) Lifespan curves of N2 wild type and N2 expressing *klf-1* under gut specific promoter (*klf-1* OE). *isp-1(qm150);ctb-1(qm189)* animals were used as controls. (**d**) mRNA expression levels of *cyp-13a7*, *cyp-14a1* and *cyp-13a11* were assayed by qPCR in *klf-1* overexpressing N2 animals (*klf-1* OE) at D1. Data are presented as mean±SEM. n=5 samples per condition. (**e**) Lifespan curves of *klf-1* overexpressing strain grown either on control (EV) or combined *cyp-25a1* and *cyp-13a11* RNAi (*cyps*). (**f**) N2 wild type and N2 overexpressing *klf-1* were grown on control (EV) and combined *cyp-25a1* and *cyp-13a11* RNAi (*cyps*) and treated with 20mM H_2O_2 at D1. Survival was assayed 4 hours after treatment. *p<0.05, ***p<0.001 One-way ANOVA with Tukey post hoc test. n=100 animals per condition.

Supplementary Table 1. GO terms of the gene list in Supplementary Data 1.

GO-ID	Pathway	P-value
55114	oxidation reduction	6.60E-04
30258	lipid modification	1.80E-02
6635	fatty acid beta- oxidation	8.80E-02
9062	fatty acid catabolic processes	8.80E-02
19395	fatty acid oxidation	9.70E-02
34440	lipid oxidation	9.70E-02

Supplementary Table 2. KEGG pathways of the gene list in Supplementary Data 1.

Pathway	P-value
Metabolism of xenobiotics by cytochrome P450	1.70E-03
Drug metabolism	2.40E-03
Fatty acid metabolism	1.50E-02
Glycolysis / Gluconeogenesis	4.60E-02
