# Supplementary material for: KLF-1 orchestrates a xenobiotic detoxification program essential for longevity of mitochondrial mutants
Source: Nat Commun. 2019 Jul 25;10:3323. doi: 10.1038/s41467-019-11275-w (PMC6658563; doi:10.1038/s41467-019-11275-w)
Supplement: Supplementary file 3 — Description of Additional Supplementary Files [file 41467_2019_11275_MOESM3_ESM.pdf]

Description of Additional Supplementary Files

File Name: Supplementary Data 1
Description: List of genes which expression is significantly changed in *isp-1;ctb-1* mutant and is not affected by developmental *klf-1* RNAi, but is brought to wild type levels by *klf-1* RNAi in adulthood.

File Name: Supplementary Data 2
Description: CA/GCCC sites in the promoters of genes listed in Figure 4a and *cyp* genes that are mostly upregulated in *isp-1(qm150);ctb1(qm189)* in *klf-1* dependent manner.

File Name: Supplementary Data 3
Description: List of genes identified in the ChiP-Seq assay, that overlap between N2 and *isp-1(qm150);ctb-1(qm189).*

File Name: Supplementary Data 4
Description: Compilation of data for lifespan experiments in Figures 1, 5, 6 and 7 and Supplementary Figures 1, 3, 5, 6 and 7.

File Name: Supplementary Data 5
Description: List of primers used in qPCR experiments in this study.
