# Supplementary material for: KLF-1 orchestrates a xenobiotic detoxification program essential for longevity of mitochondrial mutants
Source: Nat Commun. 2019 Jul 25;10:3323. doi: 10.1038/s41467-019-11275-w (PMC6658563; doi:10.1038/s41467-019-11275-w)
Supplement: Supplementary file 9 — Reporting Summary [file 41467_2019_11275_MOESM9_ESM.pdf]

nature research

Corresponding author(s): NCOMMS-18-18503

Last updated by author(s): Jun 5, 2019

Reporting Summary

Nature Research wishes to improve the reproducibility of the work that we publish. This form provides structure for consistency and transparency in reporting. For further information on Nature Research policies, see Authors & Referees and the Editorial Policy Checklist.

Statistics

For all statistical analyses, confirm that the following items are present in the figure legend, table legend, main text, or Methods section.

n/a	Confirmed	
☐	☒	The exact sample size (*n*) for each experimental group/condition, given as a discrete number and unit of measurement
☐	☒	A statement on whether measurements were taken from distinct samples or whether the same sample was measured repeatedly
☐	☒	The statistical test(s) used AND whether they are one- or two-sided<br>*Only common tests should be described solely by name; describe more complex techniques in the Methods section.*
☐	☒	A description of all covariates tested
☐	☒	A description of any assumptions or corrections, such as tests of normality and adjustment for multiple comparisons
☐	☒	A full description of the statistical parameters including central tendency (e.g. means) or other basic estimates (e.g. regression coefficient) AND variation (e.g. standard deviation) or associated estimates of uncertainty (e.g. confidence intervals)
☐	☒	For null hypothesis testing, the test statistic (e.g. *F*, *t*, *r*) with confidence intervals, effect sizes, degrees of freedom and *P* value noted<br>*Give P values as exact values whenever suitable.*
☒	☐	For Bayesian analysis, information on the choice of priors and Markov chain Monte Carlo settings
☒	☐	For hierarchical and complex designs, identification of the appropriate level for tests and full reporting of outcomes
☒	☐	Estimates of effect sizes (e.g. Cohen's *d*, Pearson's *r*), indicating how they were calculated

Our web collection on statistics for biologists contains articles on many of the points above.

Software and code

Policy information about availability of computer code

Data collection	Code for CHIP-seq data analysis is provided here:<br>https://bmcgenomics.biomedcentral.com/articles/10.1186/s12864-015-1695-x
Data analysis	Database for Annotation, Visualization and Integrated Discovery (DAVID), version 6.7<br>ImageJ 1.43<br>DatLab4 Software 4.3<br>Excel2016<br>GraphPrism 5<br>MACS2 version 2.0.10

For manuscripts utilizing custom algorithms or software that are central to the research but not yet described in published literature, software must be made available to editors/reviewers. We strongly encourage code deposition in a community repository (e.g. GitHub). See the Nature Research guidelines for submitting code & software for further information.

Data

Policy information about availability of data

All manuscripts must include a data availability statement. This statement should provide the following information, where applicable:

- Accession codes, unique identifiers, or web links for publicly available datasets
- A list of figures that have associated raw data
- A description of any restrictions on data availability

The source data underlying this study are provided as a Source Data file or available from the authors upon reasonable request. Microarray and CHIP-seq data are available on NCBI Gene Expression Omnibus (GEO) under accession number GSE61771 for Microarray and GSE130035 for CHIP-seq.

Field-specific reporting

Please select the one below that is the best fit for your research. If you are not sure, read the appropriate sections before making your selection.

☒ Life sciences ☐ Behavioural & social sciences ☐ Ecological, evolutionary & environmental sciences

For a reference copy of the document with all sections, see nature.com/documents/nr-reporting-summary-flat.pdf

Life sciences study design

All studies must disclose on these points even when the disclosure is negative.

Sample size	Lifespan analysis 100 worms; Fluorescence microscopy minimum 10 worms; Behavioral assays minimum 10 worms; Toxicity assays 100 worms. qPCR 3-5 independent samples of >100 worms.
Data exclusions	No.
Replication	3 replicates
Randomization	For all experiments, worms were randomly selected.
Blinding	No

Reporting for specific materials, systems and methods

We require information from authors about some types of materials, experimental systems and methods used in many studies. Here, indicate whether each material, system or method listed is relevant to your study. If you are not sure if a list item applies to your research, read the appropriate section before selecting a response.

Materials & experimental systems

n/a	Involved in the study	
☐	☒	Antibodies
☐	☒	Eukaryotic cell lines
☒	☐	Palaeontology
☐	☒	Animals and other organisms
☒	☐	Human research participants
☒	☐	Clinical data

Methods

n/a	Involved in the study	
☐	☒	ChIP-seq
☒	☐	Flow cytometry
☒	☐	MRI-based neuroimaging

Antibodies

Antibodies used	MnSOD (Upstate #06-984); Grp75 (Abcam #82591), HSP-60 (BD Transduction Laboratories #611562), GFP (Invitrogen #A11122), HSC70 (Santa Cruz #sc-7298), Tubulin (Calbiochem #CP06), GFP-Trap (Chromotek #gtma20)
Validation	Antibodies are validated by manufacturer and in this study.

Eukaryotic cell lines

Policy information about cell lines

Cell line source(s)	Hepa1-6 cells (ATCC-CRL-1830)
Authentication	The cell lines used have been authenticated by ATCC
Mycoplasma contamination	Negative for mycoplasma contamination
Commonly misidentified lines (See ICLAC register)	No commonly misidentified lines were used.

Animals and other organisms

Policy information about studies involving animals; ARRIVE guidelines recommended for reporting animal research

Laboratory animals	C. elegans

Wild animals	No
Field-collected samples	No
Ethics oversight	No

Note that full information on the approval of the study protocol must also be provided in the manuscript.

ChIP-seq

Data deposition

☒ Confirm that both raw and final processed data have been deposited in a public database such as GEO.

☒ Confirm that you have deposited or provided access to graph files (e.g. BED files) for the called peaks.

Data access links
May remain private before publication.

https://www.ncbi.nlm.nih.gov/geo/query/acc.cgi?acc=GSE130035

Files in database submission

K002000300_100000_S71_L008_R1_001.fastq.gz
K002000300_100001_S72_L008_R1_001.fastq.gz
K002000300_100002_S73_L008_R1_001.fastq.gz
K002000300_100003_S74_L008_R1_001.fastq.gz
K002000300_100004_S75_L008_R1_001.fastq.gz
K002000300_100005_S76_L008_R1_001.fastq.gz
K002000300_100006_S77_L008_R1_001.fastq.gz
K002000300_100007_S78_L008_R1_001.fastq.gz
K002000300_100008_S79_L008_R1_001.fastq.gz
K002000300_100009_S80_L008_R1_001.fastq.gz
K002000300_99998_S69_L008_R1_001.fastq.gz
K002000300_99999_S70_L008_R1_001.fastq.gz
27530.fwd.bigwig
27531.fwd.bigwig
27532.fwd.bigwig
27536.fwd.bigwig
27537.fwd.bigwig
27538.fwd.bigwig
27533.fwd.bigwig
27534.fwd.bigwig
27535.fwd.bigwig
27539.fwd.bigwig
27540.fwd.bigwig
27541.fwd.bigwig
27530.rev.bigwig
27531.rev.bigwig
27532.rev.bigwig
27536.rev.bigwig
27537.rev.bigwig
27538.rev.bigwig
27533.rev.bigwig
27534.rev.bigwig
27535.rev.bigwig
27539.rev.bigwig
27540.rev.bigwig
27541.rev.bigwig
1189457.narrow.bigbed
1189458.narrow.bigbed
1189459.narrow.bigbed
1189457.broad.bigbed
1189458.broad.bigbed
1189459.broad.bigbed
1189460.narrow.bigbed
1189461.narrow.bigbed
1189462.narrow.bigbed
1189460.broad.bigbed
1189461.broad.bigbed
1189462.broad.bigbed

Genome browser session
(e.g. UCSC)

http://genome-euro.ucsc.edu/cgi-bin/hgTracks?db=ce10&lastVirtModeType=default&lastVirtModeExtraState=&virtModeType=default&virtMode=0&nonVirtPosition=&position=chrII%3A8851134-10744133&hgsid=231907716_hoKPiPVFbQkKzUFXbjujwsxYAohl

Methodology

Replicates	INPUT 4081 1-3 with corresponding ATR4081 1-3, INPUT ATR4082 with corresponding ATR4082 1-3. ATR4081 is N2;Is[pvha-6::klf-1-yfp] and ATR4082 is isp-1(qm150);ctb-1(qm189);Is[pvha-6::klf-1-yfp].
Sequencing depth	Total number of reads: INPUT 4081 1 27.9M, INPUT 4081 2 30.2M, INPUT 4081 3 31.8M, INPUT 4082 1 28.9M, INPUT 4082 2 33.4M, INPUT 4082 3 30.1M, 4081 1 27.4M, 4081 2 27.9M, 4081 3 25M, 4082 1 30.2M, 4982 2 32.9M, 4082 3 28.1M.<br>Uniquely mapped reads: INPUT 4081 1 27.1M, INPUT 4081 2 29.3M, INPUT 4081 3 30.2M, INPUT 4082 1 26.9M, INPUT 4082 2 32.3M, INPUT 4082 3 29.2M, 4081 1 19.8M, 4081 2 22.6M, 4081 3 19.5M, 4082 1 25.5M, 4982 2 27.2M, 4082 3 23.2M.<br>Length of reads: 35-51bp<br>Reads were single-end.
Antibodies	Antibody used is: GFP-TRAP (Chromotek; Cat. #gtma-20; Lot. 81025001MA)
Peak calling parameters	MACS2 version 2.0.10.
Data quality	Data with fold enrichment higher than 1.5 and p-value lower than 0.05 was analyzed.
Software	Data analysis details are provided here:<br>https://bmcgenomics.biomedcentral.com/articles/10.1186/s12864-015-1695-x
